# Étude anthropométrique et pelvimétrique externe chez les nullipares de Lubumbashi: facteurs de risque et score prédictif de la dystocie mécanique

**DOI:** 10.11604/pamj.2018.31.69.16014

**Published:** 2018-10-02

**Authors:** Fanny Kaj Malonga, Olivier Mukuku, Micrette Tshanda Ngalula, Prosper Kakudji Luhete, Jean-Baptiste Kakoma

**Affiliations:** 1 Département de Gynécologie-Obstétrique, Faculté de Médecine, Université de Lubumbashi, Lubumbashi, République Démocratique du Congo; 2 Institut Supérieur des Techniques Médicales de Lubumbashi, Lubumbashi, République Démocratique du Congo; 3 Polyclinique Shalina, Lubumbashi, République Démocratique du Congo

**Keywords:** Accouchement, anthropométrie, pelvimétrie, dystocie mécanique, score prédictif, Childbirth, anthropometry, pelvimetry, mechanical dystocia, predictive score

## Abstract

**Introduction:**

La morbi-mortalité maternelle et périnatale reste élevée dans la majeure partie de l'Afrique subsaharienne par rapport au reste du monde. Dépister, avant le travail, les femmes à risque de dystocies mécaniques et les référer à un hôpital mieux équipé pour accouchement contribue à la stratégie visant à réduire la morbi-mortalité. L'objectif de cette étude est de développer un score prédictif de la dystocie mécanique lors de l'accouchement chez les nullipares congolaises.

**Méthodes:**

Il s'agit d'une étude transversale analytique sur les nullipares avec des grossesses uniques dans 7 maternités de la ville de Lubumbashi (RDC). La taille, le poids et les mensurations pelvimétriques externes maternelles ont été récoltés et analysés. Une analyse univariée et une analyse multivariée étaient réalisées. La discrimination du score était évaluée à l'aide de la courbe ROC.

**Résultats:**

Nous avons recruté 535 nullipares au cours de la période d'étude, dont 126 (23,55%) avaient accouché par césarienne indiquée pour dystocie mécanique. Après modélisation logistique, trois critères sont ressortis comme facteurs prédictifs de dystocie mécanique: la taille maternelle <150cm (ORajusté=2,96 [1,49-5,87]), le diamètre bi-ischiatique <8cm (ORajusté=15,96 [3,46-73,56]) et le diamètre prépubien de Trillat <11cm (ORajusté=2,34 [1,36-4,01]). L'aire sous la courbe ROC du score est de 0,6549 avec une sensibilité de 23,81%, une spécificité de 97,80% et une valeur prédictive positive de 76,92%.

**Conclusion:**

Il a été observé que les valeurs de 10^ème^ percentile des trois mesures anthropométriques maternelles étaient prédictives de la dystocie mécanique. Lorsqu'elles étaient utilisées ensemble, ces trois valeurs permettaient de développer un score de dépistage à faible coût pour une utilisation dans les milieux à faible revenu.

## Abstract (en)

**Introduction:**

Maternal and perinatal morbi-mortality is higher in most of sub-Saharan Africa compared to the rest of the world. All women at risk for mechanical dystocia should be screened before labor and referred to a better equipped Hospital for childbirth. This would reduce morbi-mortality. This study aims to develop a predictive score of mechanical dystocia during childbirth among nulliparous Congolese women.

**Methods:**

We conducted a cross-sectional study of nulliparous women with single pregnancy in 7 Maternity Units in the city of Lubumbashi (DRC). Women’ size, weight, and external pelvimetry results were collected and analyzed. Univariate and multivariate analyses were performed. Discrimination score was assessed using the ROC curve.

**Results:**

We included in the study 535 nulliparous women, of whom 126 (23.55%) had given birth by cesarean section due to mechanical dystocia. After logistic modelling, three criteria emerged as predictive factors for mechanical dystocia: maternal height <150 cm (adjusted OR=2.96 [1,49-5,87]), bi-ischiatic diameter <8 cm (adjusted OR =15.96 [3,46-73,56]), and Trillat’s pre-pubic diameter <11 cm (adjusted OR =2.34 [1,36-4,01]). The area under the ROC curve of the score was 0.6549 with a sensitivity of 23.81%, a specificity of 97.80% and a positive predictive value of 76.92%.

**Conclusion:**

It has been observed that 10th percentile of the three maternal anthropometric measures was predictive of mechanical dystocia. When they were used together, these three values allowed for the development of lowest-cost screening score for use in low income settings.

## Introduction

La maternité sans risque constitue l'une des priorités actuelles visant à améliorer la santé maternelle et infantile. C'est ainsi que l'Organisation Mondiale de la Santé (OMS) avait adopté la surveillance du travail et la détection précoce des dystocies comme l'une des approches les plus appropriées pour réduire la morbi-mortalité maternelle et infantile [1, 2]. La morbi-mortalité maternelle et périnatale demeure élevée dans la majeure partie de l'Afrique subsaharienne par rapport au reste du monde. La mortalité néonatale constitue un problème majeur pour la plupart des pays en développement où ces décès représentent une grande proportion de la mortalité infantile des moins de 5 ans [3]. Ces proportions élevées de décès d'enfants sont associées à des taux tout aussi élevés de morbidité et de mortalités maternelles. Trois pays d'Afrique subsaharienne dont la République démocratique du Congo, le Nigéria et l'Éthiopie faisaient partie des six pays responsables de plus de 50% des décès maternels dans le monde [4]. Selon les récentes estimations, le ratio de mortalité maternelle s'établit à 846 décès pour 100.000 naissances vivantes et le taux de mortalité néonatale à 28‰ en République Démocratique du Congo (RDC) [5]. Ces taux élevés de mortalité du couple mère-enfant sont révélateurs de l'absence de suivi de grossesse ou de mauvaise qualité des soins péripartaux [6]. Une récente étude menée à Lubumbashi par Maleya avait trouvé que 21,23% des accouchées n'avaient suivi aucune consultation prénatale et que 50,29% en avaient suivi moins de 4 [7].

Une nullipare est une femme qui n'a jamais eu une expérience d'accouchement et ses performances obstétricales ultérieures dépendront de la façon dont la première parturition grossesse sera gérée [8]. L'accouchement chez la nullipare est associé à des nombreuses complications et par conséquent, la nulliparité est considérée comme étant un facteur à haut risque en raison des préoccupations maternelles et fœtales [9, 10]. Les nullipares sont sujettes aux dystocies surtout mécaniques. Selon la littérature, plus de 50% des césariennes réalisées chez les nullipares sont indiquées pour dystocie [11, 12]. Les premières grossesses sont ainsi à risque accru des complications pendant la grossesse et l'accouchement. Lorsqu'elles ne sont pas correctement suivies, ces complications peuvent entraîner une augmentation de la morbidité et de la mortalité du couple mère-enfant [10]. Le risque intrapartum chez toutes les nullipares est fondé principalement sur l'absence d'antécédents obstétricaux [11]. Dépister, au cours des consultations prénatales et/ou avant le travail, les nullipares à risque de dystocie et les référer en temps opportun à un hôpital mieux équipé pour accouchement est l'une des stratégies contribuant à la réduction de la morbidité et de la mortalité maternelles et périnatales [13]. Cela avait conduit au développement et à l'utilisation de l'imagerie médicale (échographie, imagerie par résonance magnétique) dans la pelvimétrie en tant que méthode pour améliorer la prédiction des résultats de l'accouchement [14-18] dans l'intérêt d'améliorer la qualité des soins péripartaux afin de réduire la morbi-mortalité maternelle et périnatale [19]. Par ailleurs, les pays en développement d'une manière générale et la RDC en particulier, font face à plusieurs défis telles que le plateau technique déficient, la sous-qualification du personnel œuvrant dans les maternités périphériques, l'insuffisance des services de santé ou leur mauvaise répartition, les difficultés d'accès aux structures de référence ainsi que la recrudescence des maternités périphériques de fortune et non viables [20-22].

C'est ainsi que, sur base de ces préoccupations, l'OMS avait défini les caractéristiques d'un test de dépistage idéal pour les pays à ressources limitées comme étant un test abordable, sensible, spécifique, convivial (simple à utiliser), rapide, sans équipement et livré à ceux qui en ont le plus besoin [23]. Dans cette étude, nous nous sommes intéressés aux mensurations anthropométriques et pelvimétriques externes maternelles qui sont faciles à obtenir au cours des consultations prénatales ou lors de la parturition pour nous permettre de prédire le risque de dystocie mécanique chez les nullipares dans notre contexte. Plusieurs études ont démontré que la petite taille maternelle était significativement associée à la dystocie chez les nullipares conduisant à un accouchement par césarienne [11, 24-29]. Cette étude visait à développer un score prédictif de la dystocie mécanique lors de l'accouchement dans une population des nullipares congolaises sur base des mesures anthropométriques et pelvimétriques externes.

## Méthodes

Il s'agit d'une étude transversale analytique menée chez les gestantes nullipares reçues en consultation prénatale et en salle d'accouchement de 7 institutions hospitalières choisies dans la ville de Lubumbashi au cours de la période allant du 14 février 2016 au 31 août 2017. Le personnel médical dans chacun de ces hôpitaux était capable de pratiquer une césarienne et comptait un minimum de 80 accouchements par mois. Nous avons inclus uniquement les gestantes nullipares avec grossesse monofœtale et présentation du sommet à terme sans antécédents médicaux comme l'hypertension ou le diabète et ayant donné leur consentement libre et éclairé verbalement. L'échantillonnage était exhaustif, car elles ont été recrutées chaque jour consécutivement dans chacune des maternités par une équipe de médecins et sages-femmes formés auparavant à cette fin. Pour chaque mère, nous avons recueilli des données sur l'âge (en années), la taille (en centimètres), le poids à l'admission en salle de travail (en kilogrammes mesurés), la pelvimétrie externe. Après l'accouchement, nous avons également recueilli des données sur le mode d'accouchement.

La pelvimétrie externe comprenait les diamètres (en centimètres) suivants: diamètre bi-crête (distance séparant les sommets des crêtes iliaques), diamètre interépineux (distance séparant les deux épines iliaques antéro-supérieures et faisant partie du détroit moyen), diamètre inter-trochantérien (distance séparant les deux trochanters), diamètre bi-ischiatique (faisant partie du détroit inférieur et réunissant les faces internes des deux tubérosités ischiatiques), diamètre antéropostérieur ou conjugué externe de Baudelocque (allant de l'apophyse épineuse de L5 au bord supérieur de la symphyse pubienne), diamètre promonto-rétro-pubien (qui va du promontoire au bord supérieur de la symphyse pubienne), diamètre prépubien de Trillat (distance joignant les deux plis inguinaux, au niveau du bord supérieur du pubis et constituant la base du triangle de Trillat) [30].

Le pelvimètre de Breisky était utilisé pour mesurer les diamètres bi-crête, bi-épineux, inter-trochantérien et antéro-postérieur, tandis que le mètre ruban avait servi à mesurer la base du triangle de trillat et le diamètre bi-ischiatique. La valeur du diamètre promonto-rétro-pubien, qui est un diamètre pelvien interne et devrait être considéré comme un paramètre incidentiel au regard de la présente étude, était obtenue en retranchant 9cm à la valeur du diamètre antéro-postérieur. Les valeurs anthropométriques et pelvimétriques externes utilisées comme valeurs seuils pour identifier les femmes à risque de dystocie mécanique ont été définies comme les valeurs inférieures au 10ème percentile de notre population [25]. Nous avons formé les médecins et sages-femmes à chaque site sur la façon de mesurer la pelvimétrie externe et de remplir le questionnaire de l'étude au début de celle-ci. Sur chaque site, le responsable a été recruté comme superviseur du site pendant toute la durée de l'étude pour s'assurer que tous les formulaires étaient remplis et que les mesures étaient bien prises.

Le logiciel STATA 12 a été utilisé pour les différentes analyses statistiques. Pour déterminer les facteurs prédictifs de dystocie, nous avons effectué une analyse unifactorielle en utilisant le test du Chi2 ou le test exact de Fisher avant de réaliser une analyse multifactorielle avec une régression logistique. Le test T de *Student* a été utilisé pour la comparaison des moyennes. Les variables ayant un degré de signification inférieur à 0,05 dans l'analyse unifactorielle ont été incluses dans le modèle multifactoriel en utilisant la méthode pas à pas. Nous avons retenu dans le modèle final les variables dont le seuil de signification était inférieur à 0,05.

La discrimination du score était évaluée à l'aide de la courbe ROC et du C-index et la calibration du score selon le test d'*Hosmer-Lemeshow*. Nous avons déterminé la sensibilité, la spécificité et le pourcentage de cas correctement classés par rapport au C-index. L'évaluation de la robustesse des coefficients du modèle était faite par robust. Le score prédictif du risque était déduit au terme de l'analyse statistique et établi en assignant des points à chaque facteur de risque retenu dans le modèle logistique. Pour le rendre simple à utiliser, le score était réalisé par l'utilisation de valeurs arrondies de ces coefficients. Les probabilités de risque de dystocie en fonction des valeurs du score construit ont été calculées. L'approbation éthique a été obtenue auprès du Comité d'éthique médicale de l'Université de Lubumbashi. Un consentement oral libre et éclairé de toutes les personnes impliquées dans cette étude a été obtenu verbalement. L'anonymat a été respecté et les autorisations des médecins directeurs avaient été obtenues préalablement.

## Résultats

Nous avons recruté 535 nullipares au cours de la période d´étude, dont 1^26^ (23,55%) avaient accouché par césarienne indiquée pour dystocie mécanique. Nous avons établi la distribution des valeurs anthropométriques et pelvimétriques des 535 nullipares; seuls les 5ème, 10ème et 25ème percentiles de ces valeurs sont présentés dans le Tableau 1. Les valeurs utilisées comme valeurs seuils pour identifier les femmes à risque de dystocie mécanique ont été définies comme les valeurs inférieures au 10ème percentile de notre échantillon d'étude. Le Tableau 2 présente l'analyse univariée de la prédiction de la dystocie mécanique en fonction des mesures anthropométriques et de la pelvimétrie externe. Des 51 nullipares mesurant <150cm, 31 (60,78%) avaient présenté une dystocie mécanique contre 95 des 484 (19,63%) mesurant 150cm ou plus. On a noté une différence très hautement significative en défaveur de celles mesurant moins de 150cm (p<0,00001), traduisant un risque de dystocie de 6,32 fois plus élevé chez celles-ci (OR=6,32 [3, 46-11, 74]). Concernant le diamètre bi-crête, des 29 nullipares ayant un diamètre bi-crête <22cm, 12 (41,38%) avaient présenté une dystocie mécanique contre 114 des 506 (22,53%) ayant un diamètre mesurant 22cm ou plus. Une différence significative en défaveur de celles ayant un diamètre bi-crête <22cm (p=0,0201) a été notée, traduisant un risque de dystocie de 2,43 fois plus élevé chez celles-ci (OR=2,43 [1, 13-5, 23]). Quant au diamètre inter-épineux, des 41 nullipares ayant un diamètre inter-épineux <20cm, 19 (46,34%) avaient présenté une dystocie mécanique contre 107 des 494 (21,66%) ayant un diamètre mesurant 20cm ou plus. On a noté une différence très hautement significative en défaveur de celles ayant un diamètre inter-épineux <20cm (p=0,0003), traduisant un risque de dystocie de 3,12 fois plus élevé chez celles-ci (OR=3,12 [1, 63-5, 98]).

**Tableau 1:** 5ème, 10ème et 25ème percentiles des mensurations anthropométriques et pelvimétriques de 535 nullipares incluses dans l’étude

Variable	5^ème^percentile	10^ème^percentile	25^ème^percentile
Taille	146 cm	150 cm	154 cm
Bi-crête	21 cm	22 cm	24 cm
Diamètre bi-épineux	19 cm	20 cm	21 cm
Diamètre inter-trochantérien	25 cm	26 cm	28 cm
Diamètre bi-ischiatique	8 cm	8 cm	9 cm
Diamètre antéro-postérieur	17 cm	18,1 cm	19 cm
Diamètre promonto-retro-pubien	8 cm	9,1 cm	10 cm
Base de Trillat	11 cm	11 cm	12 cm
Poids	49 cm	52 cm	57 cm

**Tableau 2:** Analyse univariée de la prédiction de la dystocie mécanique en fonction des mesures anthropométriques et de la pelvimétrie externe

Variable	Total (N=535)	Dystocie (n=126)		Eutocie (n=409)		OR brut [IC95%]	p
		n	%	n	%		
**Taille**							
<150 cm	51	31	60,78	20	39,22	6,32 [3,46-11,74]	<0,00001
≥150 cm	484	95	19,63	389	80,37	1,00	
**Bi-crête**							
< 22 cm	29	12	41,38	17	58,62	2,43 [1,13-5,23]	0,0201
≥ 22 cm	506	114	22,53	392	77,47	1,00	
**Inter-épineux**							
< 20 cm	41	19	46,34	22	53,66	3,12 [1,63-5,98]	0,0003
≥ 20 cm	494	107	21,66	387	78,34	1,00	
**Bi-trochantérien**							
< 26 cm	46	24	52,17	22	47,83	4,13 [2,23-7,68]	<0,00001
≥ 26 cm	489	102	20,86	387	79,14	1,00	
**Bi-ischiatique**							
< 8 cm	20	18	90,00	2	10,00	33,91 [7,75-148,43]	<0,0001
≥ 8 cm	515	108	20,97	407	79,03	1,00	
**Antéro-postérieur**							
< 18,1 cm	84	66	78,57	18	21,43	23,89 [13,27-43,01]	<0,0001
≥ 18,1 cm	451	60	13,30	391	86,70	1,00	
**Promonto-retro-pubien**							
< 9,1 cm	90	68	75,56	22	24,44	20,62 [11,84-35,90]	<0,0001
≥ 9,1 cm	445	58	13,03	387	86,97	1,00	
**Trillat**							
< 11 cm	86	39	45,35	47	54,65	3,45 [2,12-5,60]	<0,0001
≥ 11 cm	449	87	19,38	362	80,62	1,00	
**Poids**							
< 52 cm	49	20	40,82	29	59,18	2,47 [1,34-4,54]	0,0028
≥ 52 cm	443	106	21,81	380	78,19	1,00	

Des 46 nullipares ayant un diamètre bi-trochantérien <26cm, 24 (52,17%) avaient présenté une dystocie mécanique contre 102 des 489 (20,86%) ayant un diamètre mesurant 26cm ou plus. Il y avait une différence très hautement significative en défaveur de celles ayant un diamètre bi-trochantérien <26cm (p<0,0001), traduisant un risque de dystocie de 4,13 fois plus élevé chez celles-ci (OR=4,13 [2, 23-7,68]). Des 20 nullipares ayant un diamètre bi-ischiatique <8cm, 18 (90,00%) avaient présenté une dystocie mécanique contre 108 des 515 (20,97%) ayant un diamètre mesurant 8cm ou plus. On a noté une différence très hautement significative en défaveur de celles ayant un diamètre bi-ischiatique <8cm (p<0,0001), traduisant un risque de dystocie de 33,91 fois plus élevé chez celles-ci (OR=33,91 [7, 75-148, 43]). Des 84 nullipares ayant un diamètre antéro-postérieur <18,1cm, 66 (78,57%) avaient présenté une dystocie mécanique contre 60 des 451 (13,30%) ayant un diamètre mesurant 18,1cm ou plus. Il y avait une différence très hautement significative en défaveur de celles ayant un diamètre antéro-postérieur <18,1cm (p<0,0001), traduisant un risque de dystocie de 23,89 fois plus élevé chez celles-ci (OR=23,89 [13, 27-43, 01]). Des 90 nullipares ayant un diamètre promonto-rétro-pubien <9,1cm, 68 (75,56%) avaient présenté une dystocie mécanique contre 58 des 445 (13,03%) ayant un diamètre mesurant 9,1cm ou plus. Une différence très hautement significative a été observée en défaveur de celles ayant un diamètre promonto-rétro-pubien <9,1cm (p<0,0001), traduisant un risque de dystocie de 20,62 fois plus élevé chez celles-ci (OR=20,62 [11, 84-35, 90]). Des 90 nullipares ayant un diamètre prépubien de Trillat <11cm, 68 (75,56%) avaient présenté une dystocie mécanique contre 58 des 445 (13,03%) ayant un diamètre mesurant 11cm ou plus. Une différence très hautement significative a été observée en défaveur de celles ayant un diamètre prépubien de Trillat <11cm (p<0,0001), traduisant un risque de dystocie de 20,62 fois plus élevé chez celles-ci (OR=20,62 [11, 84-35, 90]). Des 49 nullipares pesant <52kg, 20 (40,82%) avaient présenté une dystocie mécanique contre 87 des 449 (19,38%) pesant 52kg ou plus. Il y avait une différence très significative en défaveur de celles ayant un poids <52kg (p=0,00^28^), traduisant un risque de dystocie de 2,47 fois plus élevé chez celles-ci (OR=2,47 [1, 34-4, 54]).

**Figure 1:**
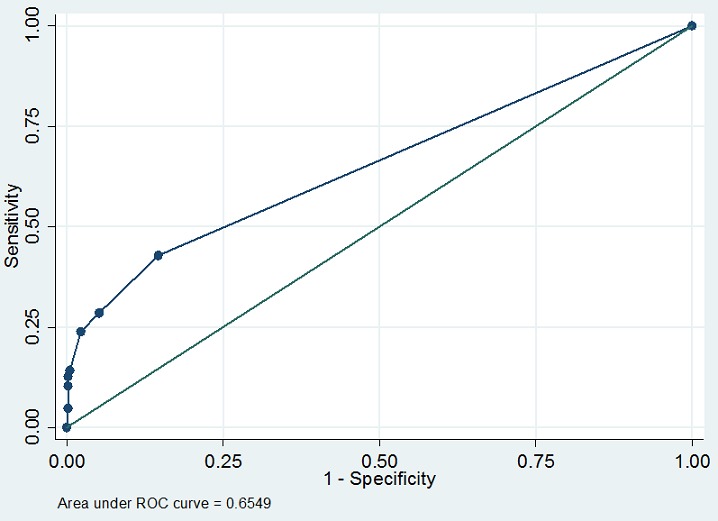
Courbe ROC du score prédictif de dystocie mécanique chez les nullipares

L'âge maternel moyen ne différait pas entre les deux groupes (p=0,4361). Les moyennes de la taille, du poids et des mensurations pelviennes étaient significativement (p<0,0001) plus petites dans le groupe de dystocie mécanique que dans le groupe d'eutocie (Tableau 3). Après modélisation logistique, une taille <150cm (OR ajusté=2,96 [1, 49-5, 87]), un diamètre bi-ischiatique <8cm (OR ajusté=15,96 [3, 46-73,56]) et un diamètre prépubien de Trillat <11cm (OR ajusté=2,34 [1, 36-4, 01]) sont ressortis comme facteurs prédictifs de dystocie (Tableau 4). Chaque facteur de risque a été pondéré par un coefficient derégression représentant le poids de la variable dans le calcul duscore. L'ensemble des scores obtenus est illustré dans le Tableau 4. Le score prédictif de la dystocie chez la nullipare a été construit à partir du modèle logistique (Tableau 4). L'aire sous la courbe ROC est de 0,6549 (Figure 1), laquelle courbe montre une discrimination acceptable en ce qui concerne sa capacité de discriminer les nullipares qui vont présenter une dystocie mécanique de celles qui ne vont pas la présenter. La présence de ces trois critères affecte un certain nombre de points dont le total est de 5 points. Pour chaque nullipare, le score varie de 0 à 5; plus il est élevé, plus le risque de dystocie est élevé. Les probabilités de risque de survenue de dystocie mécanique au cours de la parturition chez une nullipare en fonction des valeurs du score construit ont été calculées et sont présentées dans le Tableau 5. Un sore <2 définit les nullipares à faible risque de dystocie, un score entre 2 et 3 points définit un risque modéré de dystocie et un score >3 points présente un risque élevé de dystocie. Ainsi pour ce score, une sensibilité de 23,81% à été obtenue pour une spécificité de 97,80%. La valeur prédictive positive était de 76,92% et la valeur prédictive négative de 80,65% (Tableau 6).

**Tableau 3:** Comparaison des moyennes (écart-type) de l’âge et des valeurs anthropométriques et pelvimétriques de 535 nullipares

Variable	Total(N=535)	Dystocie(n=126)	Eutocie(n=409)	p
Age	23,87 (5,70)	24,25 (6,52)	23,75 (5,42)	0,4361
Taille	158,70 (7,49)	154,02 (7,27)	160,14 (6,95)	<0,0001
Poids	63,11 (9,18)	60,13 (9,79)	64,03 (8,80)	0,0001
Bi-ischiatique	9,96 (1,34)	9,06 (1,44)	10,24 (1,17)	<0,0001
Promonto-retro-pubien	11,11 (1,99)	9,35 (1,76)	11,65 (1,74)	<0,0001
Trillat	13,12 (1,84)	12,31 (1,74)	13,38 (1,80)	<0,0001
Antéro-postérieur	20,13 (2,01)	18,35 (1,76)	20,68 (1,74)	<0,0001
Inter-épineux	22,67 (2,29)	21,80 (1,99)	22,94 (2,31)	<0,0001
Bi-crête	25,20 (2,16)	24,14 (1,92)	25,52 (2,13)	<0,0001
Bi-trochantérien	29,14 (2,75)	27,66 (2,26)	29,59 (2,73)	<0,0001

**Tableau 4:** Modèle de régression logistique du risque de dystocie et score de facteurs prédictifs

Variable	OR ajusté	IC à 95%	Coefficient	Score
Taille< 150 cm	2,96	1,49-5,87	1,08	1
Bi-ischiatique< 8 cm	15,96	3,46-73,56	2,77	3
Trillat< 11 cm	2,34	1,36-4,01	0,85	1

**Tableau 5:** Probabilité de la dystocie en fonction du score selon le modèle de régression logistique

Score obtenu	Probabilité de la dystocie*
0	16,99%
1	28,27%
2	43,14%
3	59,36%
4	73,76%
5	84,40%

* obtenu à partir de la formule:

p=1/1 + exp (1,586 - 0,6549 x score)

**Tableau 6:** Sensibilité, spécificité et VPP de facteurs prédictifset du score de dystocie

Variable	10^ème^percentile	Sensibilité	Spécificité	Correctement classés
Taille	< 150 cm	24,60%	95,11%	78,50%
Bi-ischiatique	< 8 cm	30,95%	88,51%	74,95%
Trillat	< 11 cm	14,29%	99,51%	79,44%
Score		23,81%	97,80%	76,92%

## Discussion

Cette étude révèle que la combinaison des mesures de la taille maternelle <150cm associée à un diamètre bi-ischiatique <8cm et à un diamètre prépubien de Trillat <11cm est une méthode valable pour dépister les gestantes nullipares pendant la grossesse à risque pour la survenue de la dystocie mécanique au moment de l'accouchement. Une méta-analyse de la valeur de la taille maternelle en tant que facteur de risque de dystocie a montré que le 5^ème^ percentile avait une sensibilité de 21%, une spécificité de 95% [31]. Dans cette étude, nous avons pu obtenir une sensibilité de 24,60% et une spécificité de 95,11% à la valeur du 10ème percentile qui est de <150cm. Dans notre étude, les nullipares qui avaient une dystocie étaient plus courtes que celles qui avaient un accouchement normal. Ce constat corroborée celui de Munan qui, dans sa récente étude menée Lubumbashi (RDC), rapportait queles nullipares mesurant moins de 150cm présentaient 2,42 fois plus de risque d'accoucher par césarienne [11]. Il en est de même pour Liselele qui avait trouvé un OR ajusté=2,2 pour une taille maternelle <150cm [25]. Nos résultats confirment des études antérieures menées en RDC [25, 32], au Rwanda [24] et ailleurs [26-29] montrant que la petite taille maternelle était significativement associée à la césarienne chez les nullipares. Les gestantes ayant une petite taille étaient plus susceptibles d'avoir un plus grand risque de dystocie mais la sensibilité liée à une taille maternelle inférieure à 150cm était faible.

La taille des nullipares est proportionnellement corrélée aux mensurations pelvimétriques externes. Dans notre étude, les mesures pelvimétriques externes étaient significativement plus petites chez les nullipares ayant présenté une dystocieà l'accouchement que chez celles ayant eu un accouchement vaginal sans complication. Ce constat corroboré celui fait par Kakoma au Rwanda [24, 33]. Cependant, la combinaison de ces différents diamètres n'a pas entraîné une meilleure prédiction de la dystocie comme le confirme les travaux de Rozenholc [13] et ceux de Liselele [25]. Selon Bisig, les tests de dépistage fonctionnent mieux lorsqu'ils sont utilisés ensemble pour prendre des décisions sur la prise en charge des patients [34]. C'est pourquoi la taille, à elle seule, ne suffisait pas pour prédire la dystocie. L'utilité clinique de la taille maternelle est améliorée en l'associant à d'autres paramètres pour le dépistage. C'est ainsi que ce score intègre deux autres variables: les diamètres bi-ischiatique et prépubien de Trillat. Ceci signifie qu'une décision sur la prise en charge d'une mère reposera sur les résultats deces trois paramètres utilisés simultanément comme un ensemble pour l'évaluation lors d'un seul examen. Il est intéressant de rappeler que les deux diamètres pelviens concernés, qui se rapportent respectivement aux détroits inférieur et moyen du bassin obstétrical, étaient les seuls à être significativement associés à la césarienne et à la disproportion céphalo-pelvienne dans d'autres études susmentionnées [24, 33].

La dystocie est un facteur de risque majeur de morbi-mortalité maternelle et périnatale. La capacité à prédire l'échec de l'accouchement par voie basse, en dehors des facteurs intrapartum, a toujours été la principale préoccupation des sages-femmes, des médecins et des obstétriciens pratiquants dans la salle de travail. Une prédiction précise des cas à risque de dystocie permettrait de procéder à une césarienne élective et une césarienne d'urgence dans de meilleures conditions ou le plus souvent, dans notre environnement, à un transfert précoce vers les hôpitaux de district pour une surveillance de la parturition dans des conditions sûres. Si la dystocie n'est pas prédite par les centres de santé qui ne sont pas équipés pour pratiquer une césarienne, de longues distances de référence et un mauvais transport local peuvent entraîner des complications intrapartales pouvant conduire à une rupture utérine [25]. Il est vrai que s'agissant d'un test de dépistage, sa sensibilité est une fonction inverse du nombre des faux négatifs. La disproportion céphalo-pelvienne, qui est l'indication majeure en cas de bassin limite ou de bassin rétréci, est le résultat de la confrontation entre la taille de la tète fœtale et celle du bassin maternel. Sans doute que l'inclusion d'un paramètre clinique présageant les dimensions de la tète fœtale pourrait contribuer à réduire le nombre des faux négatifs et accroitre ainsi la sensibilité du test. Cette éventualité, qui n'a pas été prise en compte et qui représente une limite pour la présente étude, avait déjà été implicitement évoquée par l'un des auteurs susmentionnés [24]. Cependant, la prédiction de la dystocie chez les nullipares à risque doit être suffisamment spécifique pour éviter toute référence inutile. D'où le besoin d'un score prédictif pour guider le dépistage et le processus de prise de décision éventuel sur l'endroit où les gestantes vont accoucher. Cela signifie que les décisions basées sur la cotation de paramètres du score qui utilise des mesures anthropométriques et pelviennes faciles à prendre comme proposé dans cet article peuvent faire la différence entre la vie et la mort pour le couple mère-enfant. C'est ce qui fait que le personnel de santé, dans les milieux à faibles ressources où l'on observe la plus grande partie de la morbi-mortalité maternelle et périnatale, soit l'utilisateur idéal pour ce score.

Notre étude montre que le score clinique obtenu par la sommation de ces trois mesures s'est avéré être le meilleur modèle pour identifier les nullipares à risque de dystocie. Bien qu'il soit faiblement sensible, ce score présente une spécificité très élevée (97,80%) et permettra ainsi de mieux dépister les nullipares à risque de dystocie à l'accouchement et de minimiser le risque de faux positifs. L'avantage du score proposé est que les mesures peuvent être effectuées facilement au cours d'une visite prénatale de routine par les agents de santé, en utilisant un ruban à mesurer simple et largement disponible. Les résultats de notre étude devraient être validés prospectivement dans une cohorte distincte avant de les mettre en pratique dans la pratique courante. Ce score, après avoir été validé dans d'autres populations, pourra être très utile dans les cliniques prénatales périphériques pour identifier les femmes enceintes à risque de dystocie et les référer aux hôpitaux de généraux de référence avant le début du travail.

## Conclusion

Cette étude a identifié les diamètres bi-ischiatique et prépubien de Trillat comme deux mensurations pelvimétriques significatives et supplémentaires pour l'utilisation dans le dépistage de la nullipare à risque dedystocie mécanique lors de l'accouchement. Elle a permis à établir un score prédictif de la dystocie, lequel score comprend la taille maternelle (<150cm) associée au diamètre bi-ischiatique (<8cm) et au diamètre prépubien de Trillat (<11cm). Bien qu'il soit faiblement sensible, ce score présente une spécificité très élevée. Des études supplémentaires sont nécessaires pour évaluer davantage ce score afin de dépister les nullipares à risque dans les milieux à faible revenu. Ce score prédictif serait un outil clinique utile et simple pour limiter les taux élevés de morbi-mortalité maternelle et périnatale enregistrés dans les pays en développement, tout en recommandant que des études ultérieures puissent en augmenter la fiabilité.

### Etat des connaissances actuelles sur le sujet

- La République Démocratique du Congo (RDC) fait partie des pays comptant des taux élevés de mortalitésmaternelle et périnatale et les complications liées à l'accouchement sont l'une des principales causes de décès;
- Des études menées en RDCet ailleurs en Afrique Centrale avaient déjà identifié des seuils des mesures anthropométriques et pelvimétriques fortement associées à la disproportion céphalo-pelvienne et à une prévalence élevée de césarienne chez les nullipares. Ces mêmes études avaient recommandé des recherches approfondies en vue de mettre au point un outil de dépistage simple dans un environnement à ressources limitées.

### Contribution de notre étude à la connaissance

- L'étude proposée est la première intégrant une analyse multivariée permettant d'identifier les facteurs prédictifs de dystocie mécanique chez les nullipares dans notre contexte, à Lubumbashi, République Démocratique du Congo;
- Elle est également la première à proposer un outil trouvant son importance dans son utilisation dans le dépistage de risque de dystocie mécanique à l'accouchement dans la population de nullipares dans notre contexte.

## Conflits d’intérêts

Les auteurs ne déclarent aucun conflit d'intérêts.
